# Genetics of clozapine-associated neutropenia: recent advances, challenges and future perspective

**DOI:** 10.2217/pgs-2018-0188

**Published:** 2019-02-15

**Authors:** Sophie E Legge, James TR Walters

**Affiliations:** 1 MRC Centre for Neuropsychiatric Genetics & Genomics, Division of Psychological Medicine & Clinical Neurosciences, School of Medicine, Cardiff University, Cardiff, CF24 4HQ, UK

**Keywords:** agranulocytosis, clozapine, neutropenia, pharmacogenetics, schizophrenia

## Abstract

Clozapine is the only effective antipsychotic for treatment-resistant schizophrenia but remains widely under prescribed, at least in part due to its potential to cause agranulocytosis and neutropenia. In this article, we provide an overview of the current understanding of the genetics of clozapine-associated agranulocytosis and neutropenia. We now know that the genetic etiology of clozapine-associated neutropenia is complex and is likely to involve variants from several genes including *HLA-DQB1*, *HLA-B* and *SLCO1B3/SLCO1B7*. We describe recent findings relating to the Duffy-null genotype and its association with benign neutropenia in individuals with African ancestry. Further advances will come from sequencing studies, large, cross-population studies and in understanding the molecular mechanisms underlying these associations.

Clozapine is the only effective antipsychotic for treatment-resistant schizophrenia (TRS), one of the most disabling forms of mental illness characterized by a failure to respond to at least two antipsychotic trials of sufficient dose and duration [1–4]. Nonetheless, clozapine’s use has been limited by the potential for adverse effects and it remains widely under prescribed [5]. Of particular importance is the rare, but potentially fatal, hematological side effect of agranulocytosis: a deficiency of neutrophils (a type of granulocytic white blood cell) to levels below 500/mm^3^ that renders the person vulnerable to infections. Neutropenia is a less severe form characterized by neutrophil levels falling below 1500/mm^3^. The cumulative prevalence of agranulocytosis and neutropenia in those taking clozapine is approximately 0.9 and 3.8%, respectively, and the peak incidence is one month after exposure to clozapine treatment [6–9].

The occurrence of agranulocytosis led to clozapine being withdrawn in the 1970s after reports of associated fatalities in Finland [10,11]. However, two landmark clinical trails [1,12] demonstrated clozapine’s therapeutic superiority over chlorpromazine in patients with TRS and led to its reintroduction in the 1990s. In most countries, a condition of clozapine’s licensing was mandatory hematological monitoring to facilitate the early detection of neutropenia and thus prevent agranulocytosis. If neutropenia is identified in the UK, clozapine treatment is immediately discontinued and usually hematological parameters will subsequently normalize. However, this frequent hematological monitoring limits the acceptability of clozapine to patients and poses an additional obstacle to clinician prescribing [13].

The causes of clozapine-induced agranulocytosis (CIA) and neutropenia (CIN) remain unclear although a genetic contribution has been established [14–16]. The identification of genetic factors that increase susceptibility for agranulocytosis and neutropenia has received substantial interest as, in addition to providing etiological insights, a sensitive and reliable predictor could conceivably be used to identify patients most at risk. Furthermore, for those at low risk there could be a reduced need of regular monitoring, lessening the burden of testing on patients and services and potentially broadening the availability of clozapine. In this article, we provide an overview of the current understanding of the genetics of CIA and CIN. There have been several excellent reviews examining previous candidate gene studies [17–19], and thus the focus of this article is to describe the advances made from recent large-scale genomic studies, discuss the current challenges of this field and provide an indication of its future direction.

## Theoretical mechanisms

The mechanism by which clozapine induces agranulocytosis and neutropenia is still not well understood [20,21]. There is evidence indicating it may be caused by the bioactivation of clozapine or a stable metabolite of clozapine to a chemically reactive nitrenium ion [20,22,23], which has been shown to cause dose-dependent apoptosis to neutrophils at therapeutic levels of clozapine [24,25] as well as toxicity to stromal cells, the precursors of neutrophils in bone marrow [26]. The dose-dependent effect of the nitrenium ion suggests that metabolism, bioavailability or other factors relating to pharmacokinetics could be related to CIA and CIN, a hypothesis supported by recent association of genetic variants in the hepatic transporter gene *SLCO1B3*. However, the relationship between clozapine dose and agranulocytosis remains unclear [27]. Neutrophils themselves can generate hypochlorous acid via the NADPH/myeloperoxidase (MPO) pathway, which is capable of oxidizing clozapine to reactive metabolites that covalently bind to neutrophils [23,28,29]. The binding of these reactive metabolites could in turn lead to agranulocytosis or neutropenia via direct toxicity or by initiating an immune mechanism, or both. An immune mechanism has long been hypothesized since a number of the characteristics of CIA mirror immune-mediated reactions including its initial timing, and the fact that it can occur more quickly and severely upon rechallenge with clozapine. The association of *HLA-B* and *HLA-DQB1* variants from genetic association studies has also added support for an immune-related mechanism [14,15].

## Genetic association studies of clozapine-associated agranulocytosis & neutropenia

The possibility of a genetic contribution to CIA was highlighted from an early stage given the observation that the incidence in Finland was 20-times higher than other European countries where the drug was widely prescribed [11]. A six-generation pedigree analysis in the 1970s failed to identify any significant genetic kinship between the affected patients in Finland, indicating that a single gene following Mendelian inheritance patterns was unlikely to be responsible [30,31]. There have been reports of concordance of CIA in monozygotic twins [32,33], although no study to date has formally assessed heritability. Until recently, the majority of genetic association studies have focused on candidate genes from the human leukocyte antigen (HLA) system, which are involved in immune response. The application of modern genomic approaches has confirmed that there is a genetic component to CIA and CIN and that the genetic aetiology is complex, likely involving several genes. In this review, we briefly describe previous candidate gene studies, summarize recent genome-wide association studies (GWAS) and exome sequencing studies, and discuss the evidence for specific genetic variants that have been implicated in CIA and CIN.

### Candidate gene studies

HLA genes located in the MHC on chromosome 6 (6p21.31-32) encode proteins that are responsible for regulating the immune system and were thus hypothesized to be involved in CIA [17]. The first case–control study in 1990 by Lieberman *et al*. in six CIA cases and 25 clozapine-treated controls identified an association with *HLA-B38* [34]. The association was also reported in further studies from the same research group [35,36]. However, independent studies with similarly sized samples reported inconsistent findings for the association of HLA-B38 with CIA [37–40]. Typing of class II alleles by Yunis *et al.* revealed associations with a number of HLA allele markers in *HLA-DRB1*, *HLA-DQB1*, *HLA-DQA1* and *HLA-DR* [36], most of which have not been independently replicated.

More consistent findings came from the role of *HLA-DQB1* in CIA. The HLA marker DQB1*0201 was reported to be overrepresented in CIA cases in studies by Amar *et al.* and Dettling *et al.* [38–40]. A candidate study of 74 genes in 2011 by Athanasiou *et al*. in 33 CIA cases and 54 controls from the USA, Russia and South Africa found associations for variants within five genes: *HLA-DQB1*, *HLA-C*, *HLA-DRD1*, *NTSR1* and *CSF2RB* [15]. Using a previously reported replication sample of 49 CIA cases and 79 controls [39–41], they found evidence for an association with *HLA-DQB1* 6672G>C (OR: 16.9; 95% CI: 3.57–109) that was present in 21.5% of the combined cases and 1.6% of combined controls [15]. The sensitivity and specificity of *HLA-DQB1* 6672G>C in the combined sample was 21.5 and 98.4%, respectively. On the basis of this study, *HLA-DQB1* 6672G>C was marketed as a predictive genetic test but its low sensitivity limited its clinical utility and it has now been withdrawn due to the low uptake [17].

One research group hypothesized that the association of different HLA types in Ashkenazi Jewish and non-Jewish CIA patients could be caused by the linkage disequilibrium (LD) of a common non-HLA genetic marker in the MHC [42]. Associations were reported in regard to *HSP-70* genes [43] and *TNF* microsatellites [42,44], but these findings are yet to be independently replicated and to date there is little evidence to suggest that these genes account for the association of *HLA* genes with CIA.

Non-immune mediated hypotheses prompted other candidate gene studies. Because neutrophils can generate hypochlorous acid via the NADPH/MPO pathway, which is capable of oxidizing clozapine to reactive metabolites such as a nitrenium ion, there have been studies investigating the role of polymorphisms that alter MPO and NADPH activity. These studies have not yielded consistent findings [45,46]. However, continuing this hypothesis that a defective oxidative mechanism leads to the formation of reactive metabolites and consequently CIA, Ostrousky *et al*. found a significant association of polymorphisms in *NQO2* in 18 CIA cases and 80 clozapine-treated controls [47]. A recent candidate study also found a significant association of the *NQO2* 1541AA polymorphism in 31 CIA cases compared with 241 clozapine-treated controls [48]. Lastly, there have been negative studies published investigating the role polymorphisms in leukocyte *Fcγ* receptors [49] and *CYP2D6*, the primary gene involved in clozapine metabolism [45].

Although there have been many candidate studies, the rare incidence of CIA has limited the availability of suitable patients and thus studies have been substantially underpowered [50]. Furthermore, there has been a considerable overlap between study samples, with the majority of candidate studies coming from four or five research groups. It has become clear in the field of schizophrenia genetics that candidate gene studies have not yielded robust insights into disease etiology given the limited knowledge of underlying mechanisms [51]. The same limitations of candidate gene approaches could be leveled at studies in CIA, although in contrast there has been a consistent association demonstrated for one immune locus in particular, *HLA-DQB1*.

### Large-scale genomic studies of clozapine-associated neutropenia

Recent advances in the field of pharmacogenomics have come from large-scale genetic association studies using modern techniques such as GWAS and exome sequencing [14,16,52,53]. GWAS is a hypothesis-free approach to investigating the contribution of common SNPs to traits or disease. GWAS genotyping arrays rely on an informative backbone of tag SNPs that capture most of the common genetic variation in the genome, and have made large-scale whole-genome genotyping affordable [54]. Given the rarity of CIA, recent studies have also investigated the contribution of large, rare copy number variation (CNVs), and protein-coding variants via whole exome sequencing or exome arrays. It is of note that recent studies have selected a broader neutropenia phenotype, not restricted to agranulocytosis. This has been a pragmatic strategy given the recent rarity of agranulocytosis due to the success of monitoring systems, and this approach relies on the assumption that the development of neutropenia whilst taking clozapine serves as a proxy (or premonitory phase) of agranulocytosis.

The first whole exome sequencing study by Tiwari *et al.* in 2013 was in 24 Finnish cases with CIN (absolute neutrophil count [ANC] <1500 cells/mm^3^) and 24 age- and sex-matched controls, and did not identify any rare, functional variants that were significantly associated after correction for multiple testing [52].

In 2014, the Clozapine-Induced Agranulocytosis Consortium (CIAC) published a comprehensive genetic analysis including GWAS, whole exome sequencing, CNVs and imputed HLA alleles in up to 163 cases with CIN (ANC < 1000 cells/mm^3^), 249 clozapine-exposed controls without neutropenia and 7970 unexposed controls [14]. They found two amino acid polymorphisms in the MHC that were independently and significantly associated with CIN at the genome-wide significance level of p < 5 × 10^-8^: *HLA-DQB1* (126Q) and *HLA-B* (158T) [14]. It should be noted the sample was not independent of samples used in earlier candidate studies that reported associations with *HLA-DQB1* and *HLA-B* [15,37].

In 2016, we published a study consisting of GWAS, exome array, CNV and imputed HLA allele analyses in the CLOZUK sample of 66 CIN cases (ANC < 1500 cells/mm^3^) and 5583 controls who were all treated with clozapine for a minimum of a year without developing ANC < 2000 cells/mm^3^, and combined associated variants in a meta-analysis with the CIAC study [16]. The discovery GWAS analysis identified two intronic SNPs that were significantly associated with CIN, but were not replicated in the CIAC sample. More promising insights came from the GWAS meta-analysis; a genome-wide significant association with CIN was found for rs149104283, an intronic SNP to transcripts of *SLCO1B3* and *SLCO1B7* [16]. The CLOZUK study also found that although no single rare variant from the exome array was associated with CIN, gene-based analyses yielded association for two genes: *UBAP2* and *STARD9* [16]. Furthermore, direct genotyping in 60 cases and 305 controls found an independent replication of the previously associated variant *HLA-DQB1* 6672G>C [15].

Saito *et al*. reported a further GWAS of 50 cases with CIN (ANC < 1500 cells/mm^3^) and 380 clozapine-exposed controls without neutropenia and 2905 unexposed controls from the Japanese population [53]. As they detected a genome-wide significant MHC signal from the GWAS analyses, they conducted classical HLA typing and found a significant association of HLA-B*59:01 with CIN in comparison to both clozapine-exposed and unexposed controls.

In the most recent study, we conducted a GWAS analysis of lowest ANC during clozapine treatment (as opposed to neutropenia case vs controls) in 552 individuals from the UK with robustly inferred African ancestry [55]. Two genome-wide significant loci were identified, of which the most significant locus was indexed by rs2814778, a regulatory variant in *ACKR1* that has previously been associated with lower neutrophil counts in individuals of African ancestry, and is thought to be causal for benign ethnic neutropenia (BEN).

The strength of the evidence and clinical relevance for the key genetic variants identified from large-scale genetic studies investigating CIN is discussed below.

## Risk variants for clozapine-associated neutropenia

Table 1 details the genetic risk variants associated with CIN and CIA from large-scale common and rare variant association studies.

**Table 1.** **Genetic risk variants associated with clozapine-induced agranulocytosis/neutropenia from large-scale common and rare variant association studies.**

**Gene**	**Variant**	**Effect size**	**Phenotype**	**Populations**	**Evidence from candidate studies**	**Association studies**
*HLA-DQB1*	HLA-DQB1 (126Q) HLA-DQB1 6672G>C HLA-DQB1*05:02	5.2-16.9	CIA & CIN	White European, USA, Russia and South Africa, Israeli (Jewish and non-Jewish)	Athanasiou *et al.* [15]^†^	Goldstein *et al.* [14]^†^ Legge *et al.* [16]

*HLA-B*	HLA-B (158T) HLA-B38/B39/B67	3.3	CIA & CIN	White European, USA, Russia and South Africa, Israeli (Jewish and non-Jewish)	Lieberman *et al.* [34]^†^ Yunis *et al.* [35,36]^†^ Valevski *et al.* [37]^†^ Amar *et al.* [38] (NS) Dettling *et al.* [39,40] (NS)	Goldstein *et al.* [14]^†^

	HLA-B*59:01	6.3-15.8	CIN	Japanese		Saito *et al.* [53]

*SLCO1B3/7*	rs149104283	4.32	CIN	White European	Saito *et al.* [64] (NS)	Legge *et al.* [16]

*ACKR1*	rs2814778	20.36	CIN	African ancestry		Legge & Pardiñas *et al.* [55]

Genetic risk variants associated with CIN and CIA from large-scale common and rare variant association studies. Variants were only included that had evidence of association from non-candidate genetic studies. Columns include: gene, variant name, effect size of genetic variant, phenotype the genetic variant has been associated with, populations used in studies, candidate studies and association studies. Amino acid polymorphism *HLA-DQB1* (126Q), *HLA-DQB1* 6672G>C and HLA-DQB1*05:02 are all in strong LD and likely to convey same association signal. Similarly for *HLA-B* (158T) and HLA-B38. Effect sizes are estimated from large-scale association studies only.

^†^Studies with overlapping cases.

CIA: Clozapine-induced agranulocytosis, CIN: Clozapine-induced neutropenia; HLA: Human leukocyte antigen; LD: Linkage disequilibrium.

### HLA-DQB1

There is now considerable support for the role of variants within *HLA-DQB1* in CIN and CIA. The association of this gene was initially identified by candidate gene studies [15,36,38–40] and the imputed amino acid polymorphism *HLA-DQB1* (126Q) was the most significantly associated variant from the CIAC study [14]. *HLA-DQB1* (126Q) is in strong LD with *HLA-DQB1* 6672G>C, the variant implicated in the candidate study by Athanasiou *et al*. [15]. Although the CIAC study includes cases from the earlier candidate studies that implicated *HLA-DQB1* [15,37,41], the CLOZUK study provided further replication in an independent sample for the association of *HLA-DQB1* 6672G>C [16]. Therefore, this finding represents the most robustly associated variant for CIN and CIA. However, the causal variants and biological mechanisms of how this association leads to CIN are yet to be resolved. These findings are limited to individuals of European ancestry and so it is currently unknown whether variants in *HLA-DQB1* also increase risk in other populations.

### HLA-B

There is increasing evidence for the role of variants in *HLA-B* in CIN from large-scale genomic studies. The imputed amino acid polymorphism *HLA-B* (158T) was significantly associated with CIN in the CIAC study, independent of *HLA-DQB1* (126Q), and primarily driven by the association in individuals of Ashkenazi Jewish ancestry [14]. The amino acid change at *HLA-B* (158T) corresponds to *HLA-B38*, which has also been implicated in previous candidate gene studies [34,37], although some of these samples overlap with the CIAC study and there have also been negative reports [34–40]. Further evidence for the role of *HLA-B* has come from Saito *et al*. [53], who found a significant association of HLA-B*59:01 with CIN in the Japanese population. HLA-B*59:01 is a rare (∼1–2%) variant in Japanese and east Asian populations and reference datasets indicate that it is monomorphic in other Caucasian and African populations. Saito *et al.* report that it is not compatible with the amino acid polymorphism *HLA-B* (158T) reported by Goldstein *et al.* Nonetheless, integrating association signals from different populations might help in the discovery of causative variants by leveraging on differential LD patterns between populations. As with *HLA-DQB1*, it is currently unclear how the risk variants from *HLA-B* lead to CIN, although it is presumed to be through an immune-related mechanism.

### SLCO1B3/SLCO1B7

The meta-analysis by Legge *et al*. of CLOZUK and CIAC identified a genome-wide significant association for a genetic locus indexed by rs149104283 (odds ratio [OR]: 4.32), an intronic SNP to transcripts of hepatic transporter genes *SLCO1B3* and *SLCO1B7* [16]. rs149104283 was present in 7.37% of CIN cases versus 1.52% of controls in the CLOZUK sample and 4.20% of CIN cases versus 1.67% of controls in the CIAC sample. This finding indicates a possible pharmacokinetic mechanism for CIN as the associated region encodes for liver-specific organic anion-transporter polypeptides that facilitate the uptake of drugs such as clozapine from the portal vein into hepatocytes, where it is subsequently metabolized or excreted [56,57]. Polymorphisms from these genes have also been implicated in adverse reactions of other drugs such as docetaxel-induced neutropenia [58–61] and simvastatin-induced myopathy [62,63]. Replication of rs149104283 in independent samples and further functional studies are required to determine if these hepatic transporter genes do in fact influence the uptake or metabolism of clozapine and how this consequently leads to neutropenia and agranulocytosis.

Saito *et al*. reported a lack of replication of this signal in their sample of 50 cases with CIN and 2905 healthy control subjects from the Japanese population [64]. However, the index SNP from Legge *et al*. rs149104283, was monomorphic in their Japanese sample and the SNP they used for replication (rs11045434) was in linkage equilibrium with rs149104283 (R^2^ = 0, estimated from LDlink for all populations [65]). When the index SNP is not available, valid replication candidate SNPs must be both in high LD and within the local genomic region [66]. Thus, the use of this SNP for replication is inappropriate and does not provide evidence either for or against the association of rs149104283. Saito *et al*. selected this SNP as it was in the genomic region of *SLCO1C1* and upstream of *SLCO1B3* and showed a modest association with clozapine-associated neutropenia. It is possible that the associated regions from these two studies differ due to population differences but impact onto a common mechanism and further studies are required to investigate this possibility.

There has been a recent interest in a pharmacokinetic mechanism via polymorphisms within *ABCB1*, the P-glycoprotein transporter gene responsible for the excretion of major clozapine metabolites, as there is evidence they effect clozapine plasma levels [67,68]. However, there is very limited evidence to date to suggest they cause CIN or CIA [48,69].

### ACKR1

In the first genetic association study of neutrophil counts during clozapine treatment in UK individuals with African ancestry, we identified a strong association of a locus on chromosome 1 attributable to rs2814778 (p = 4.21 × 10^-21^), a regulatory variant in *ACKR1* [55]. The CC genotype at rs2814778 is also known as the Duffy-null genotype and is rare in most non-African populations [70]. However, in those of African ancestry, it has been robustly associated with neutrophil counts [71–74] and is also considered to be the cause of BEN [75], an hereditary condition characterized by low neutrophil counts which occurs in 25–50% of individuals with African or Middle Eastern ancestry [75–78]. In those with African ancestry, individuals with the Duffy-null genotype were substantially more likely to develop neutropenia whilst taking clozapine (OR: 20.36), indicating that clozapine-associated neutrophil levels in people of African ancestry are often a result of BEN rather than an adverse drug reaction. This finding indicates that there may be at least three genetic mechanisms impacting on CIN; those that impact immune reactivity, hepatic uptake (and potentially subsequent drug toxicity), and genetic factors that cause benign neutropenia unrelated to clozapine.

## Current indications for genetic testing

The genetic variants that have been identified to date for CIA and CIN as an adverse drug reaction (i.e., variants in *HLA-DQB1*, *HLA-B*, and *SLCO1B3/SLCO1B7*) do not fulfill the criteria for a predictive genetic test [79]. Table 2 details the sensitivity, specificity, positive predictive value (PPV) and negative predictive value (NPV) of a potential pharmacogenetic test using these variants to predict CIN. Across all reported variants, sensitivity values range from 11 to 36%, specificity from 89 to 98%, PPV from 5.1 to 25.9% and NPV from 96.9 to 99.7%. The highest sensitivity value reported to date was from Goldstein *et al*. [14] for the combination of *HLA-DQB1* (126Q) and *HLA-B* (158T). Nonetheless, these figures mean that less than half of the individuals who develop clozapine-associated neutropenia will have any one of the risk variants identified to date. A modeling study by Verbelen *et al.* demonstrated that high sensitivity is the critical factor for a successful genetic test for CIA [79]. Indeed, on the basis of the study by Athanasiou *et al*. [15], *HLA-DQB1* 6672G>C was marketed as a genetic test but the low sensitivity (21.5%) limited its clinical utility and it was withdrawn due to the low uptake [17]. Girardin *et al.* demonstrated that despite not being suitable for clinical application, a strategy to withdraw monitoring in those that have neither *HLA-DQB1* (126Q) or *HLA-B* (158T) risk variants, considering the risk is reduced to a level that is similar to other drugs, genetic testing would still be cost-effective in US settings providing it cost less than $700 per patient [80].

**Table 2.** **Sensitivity and specificity of genetic variants for clozapine-induced agranulocytosis/neutropenia.**

**Genetic variants**	**Sensitivity (%)**	**Specificity (%)**	**PPV (%)**	**NPV (%)**	**Study [Ref.]**
HLA-DQB1 6672G>C	21.5	98.4	5.1	99.7	Athanasiou *et al.*	[15]

HLA-DQB1 (126Q) and HLA-B (158T)	36.0	89.0	NR	NR	Goldstein *et al.*	[14]

SLCO1B3/7 rs149104283	10.9	97.6	14.0	96.9	Legge *et al.*	[16]

SLCO1B3/7 rs149104283 and HLA-DQB1 6672 G>C	29.2	90.6	9.9	97.3	Legge *et al.*	[16]

HLA-B*59:01 (CIN vs clozapine control)	24.0	95.3	17.4	96.8	Saito *et al.*	[53]

HLA-B*59:01 (CIA vs clozapine control)	31.8	95.3	6.4	99.3	Saito *et al.*	[53]

Sensitivity and specificity of using identified genetic variants for CIA and CIN as a predictive genetic test. Reported columns include: the genetic risk variant(s) used to predict CIN, sensitivity, specificity, PPV, NPV and the study. Values for HLA-B*59:01 variant were also consistent for CIA and CIN versus healthy controls reported by Saito *et al.* [53].

CIA: Clozapine-induced agranulocytosis, CIN: Clozapine-induced neutropenia; HLA: Human leukocyte antigen; NPV: Negative predictive value; NR: Not reported; PPV: Positive predictive value.

Recent findings have uncovered the potential for a genetic test to identify those at high risk of benign neutropenia that is unrelated to clozapine, but nonetheless disrupts treatment. Our study indicates that genotyping of rs2814778 to identify individuals with the Duffy-null genotype has clinical utility as an alternative strategy for the diagnosis of BEN in individuals with African ancestry, which is currently made by hematology assessment [55]. However, prior to the implementation of Duffy-null genotype testing, crucial studies are necessary to establish the safety, practicality and cost-benefits of such a test. Theoretically, individuals with the Duffy-null genotype could have revised neutrophil thresholds in line with current BEN monitoring procedures for clozapine treatment, provided they do not show signs of compromised immune function. This ability to prospectively lower neutrophil thresholds would permit more BEN patients to start clozapine at the lower baseline threshold and also avoid disruption of treatment for those who discontinue clozapine due to benign neutropenia. Approximately 80% of the UK population with African ancestry and the 64% of the African–American population in the USA would test positive for this pharmacogenetic screening test. However, the potential applications extend beyond those of self-reported African ancestry given rs2814778 is polymorphic in parts of the Middle East, south west Asia and Oceania [70].

## Conclusion & future perspective

The application of GWAS and other modern genomic techniques has led to advances in our understanding of the genetic basis of CIA and CIN. Recent studies have provided evidence for the role of variants in *HLA-DQB1*, *HLA-B* and *SLCO1B3/SLCO1B7* for CIN, and for the Duffy-null genotype on benign neutropenia in individuals with African ancestry. The latter has potential clinical utility, pending safety studies, as a genetic test to revise clozapine safety blood-monitoring criteria, raising the realistic prospect of clinically applicable pharmacogenetic testing in psychiatry. Future challenges will include the refinement of identified genome-wide association loci signals, through bioinformatic and perhaps sequencing efforts, and characterizing their functional and biological significance. We also advocate for the inclusion of diverse population samples in future studies particularly given the often greater burden of TRS in non-European populations. Finally, less than half of the individuals who develop CIN have a currently identified risk variant and thus further studies are required to determine why the individuals without these risk variants develop the rare adverse effect.

Recent studies indicate that the genetic etiology of CIN is complex and is likely to involve at least several genes. At present, it appears unlikely that this rare adverse effect is caused by a rare variant of very large effect, a rare protein-coding variant or a rare, pathogenic CNV [14,16,52], although the application of whole-genome sequencing is required to conclude this definitively. Although there is evidence for the role of variants in *HLA-DQB1*, *HLA-B* and *SLCO1B3/SLCO1B7*, the mechanism through which these variants lead to increased risk of CIN remains unclear. This is a limitation that applies more generally to GWAS findings in that many of the SNPs detected are noncoding variants with an unknown impact [81]. Thus, functional follow-up studies are required to determine the causal SNP(s) and associated biological mechanisms. Improved annotation of these signals together with recent advances in whole-genome sequencing should provide informative answers in this regard.

An on going challenge for the field will be the relatively rare incidence of CIN and even rarer incidence of CIA, which limits the availability of suitable patients and thus results in studies having small-sample sizes [50]. This has a significant impact on statistical power; the CIAC study had 99% power and the CLOZUK discovery sample 80% power to detect an odds ratio greater than 4.0 for alleles with a frequency greater than 0.10. Thus, even the recent genetic studies of CIN are underpowered to detect associations that do not have a moderate to large effect size. It is likely that there are causal variants of small effect that have not yet been detected and the field requires larger samples to determine if this is the case and to characterize the genetic architecture of CIN and CIA. Greater insights should be gained in the near future from the CRESTAR project, which aims to combines in meta-analysis all of the available CIN samples.

In order to increase sample sizes, two of the GWAS have used predominantly population controls as opposed to clozapine-treated or schizophrenia controls [14,53]. In these situations, it is important for any associations to be confirmed with clozapine-treated controls, as it is possible the variant could be associated with schizophrenia rather than CIN. Indeed, both of these GWAS found large association peaks around the MHC [14,53], which is also a predominant feature of schizophrenia GWAS analyses [82].

An important consideration is the use of a neutropenia phenotype as opposed to agranulocytosis in recent studies. It is possible that neutropenia and agranulocytosis have distinct etiologies [21]. However, agranulocytosis is now very rare due to the success of the monitoring system, which in many countries such as the UK, triggers discontinuation of clozapine as a precaution when neutropenia is detected. This means, however, that it is not possible to know which patients would go on to develop agranulocytosis and who may be experiencing a benign neutropenia. This is especially important in populations with increased rates of BEN; individuals with the genetic variant for BEN are 20-times more likely to develop neutropenia and have clozapine discontinued [55]. Neutropenia in these individuals is most likely benign and not an adverse effect of clozapine. Nonetheless, given in the field there are valid concerns that a more stringent threshold may produce more reliable results, it is important, as most studies have done, to confirm findings at more severe thresholds.

Historically, most studies of CIN have been performed in populations with European ancestry. This is changing, with recent studies in the Japanese population [53] and in individuals from the UK with African ancestry [55]. Cross-population studies are vital to determine the generalizability of risk variants or whether effects are population specific, and perhaps particularly important for the immune and metabolic systems that are highlighted by existing research. Furthermore, the ability to identify more genetic loci associated with CIN from different populations should help identify causative variants by leveraging on different LD patterns between populations [83]. However, failure to replicate associated variants in differing ethnicities and populations can arise for a number of reasons including that the initial association was a false-positive result, the replication is falsely negative due to study power or sample differences, or because the variant exerts a population-specific effect, and distinguishing between these is challenging [83]. A gold standard example of population-specific genetic risk comes from the rare adverse effect of Stevens–Johnson syndrome and toxic epidermal necrolysis from carbamazepine that is strongly associated with HLA-B*1502 in individuals of Asian descent [84] but with HLA-A*3101 in European and Japanese populations [85,86].

In conclusion, recent years have seen important advances in our understanding of the genetic causes of CIN and CIA. Future developments will come from increasing sample sizes to identify novel risk variants, conducting cross-population studies, identifying the causal variants and in applying approaches to understanding the molecular mechanisms underlying these associations. These approaches will be key to the clinical translation of the genetic risk for CIA.

**Executive summary**

**Theoretical mechanisms**

- The mechanism by which clozapine induces neutropenia (CIN) and agranulocytosis (CIA) is still unclear but there is evidence it is caused by the activation of clozapine to a chemically reactive nitrenium ion, which could lead to neutropenia via direct toxicity, or by initiating an immune mechanism, or both.
- The association of genetic variants in hepatic transporter genes *SLCO1B3* and *SLCO1B7* indicates a potential pharmacokinetic mechanism for CIA and CIN.
- The association of genetic variants in *HLA-DQB1* and *HLA-B* provides support for an immune related mechanism for CIN.

**Genetics of clozapine-associated neutropenia**

- Early candidate gene studies have implicated HLA genetic variants from the MHC.
- A whole exome sequencing study and four genome-wide association studies have now been published. Two genome-wide association studies included populations with European ancestry, the third included a Japanese population and the fourth investigated neutrophil counts in individuals with African ancestry. These studies have provided evidence for variants in in *HLA-DQB1*, *HLA-B* and *SLCO1B3/SLCO1B7* for CIN and evidence for the Duffy-null genotype in *ACKR1* for benign neutropenia during clozapine treatment.

**Key risk variants for clozapine-associated neutropenia**

- There is now considerable support for the role of the amino acid polymorphism *HLA-DQB1* (126Q) and HLA-DQB1 6672G>C (which are in high linkage disequilibrium) in CIN.
- The amino acid change at *HLA-B* (158T), which corresponds to the previously associated variant HLA-B38, has been associated with CIN in populations with European ancestry. HLA-B*59:01 has been associated with CIN in the Japanese population.
- CIN has also been associated with rs149104283 in those of European ancestry, an intronic SNP in hepatic transporter genes *SLCO1B3* and *SLCO1B7*.
- A strong association with the Duffy-null genotype, a regulatory variant in *ACKR1*, has been identified for benign neutropenia in people with African ancestry.

**Current indications for genetic testing**

- The genetic variants that have been identified to date for neutropenia as an adverse drug reaction do not currently indicate suitability for a predictive genetic test due to low sensitivity.
- Genotyping of rs2814778 (*ACKR1*) to identify individuals with the Duffy-null genotype in those with African ancestry offers potential as a pharmacogenetic test to diagnose benign ethnic neutropenia and prospectively revise clozapine safety blood monitoring criteria.

**Conclusion & future perspective**

- The genetic aetiology of CIN is complex and is likely to involve variants from several genes including *HLA-DQB1*, *HLA-B* and *SLCO1B3/SLCO1B7*.
- The Duffy-null genotype causes benign neutropenia, which is often mistaken for CIN, in individuals with African ancestry.
- Future advances will come from increasing sample sizes to identify novel risk variants, conducting cross-population studies, identifying causal variants from association signals and in understanding the molecular mechanisms underlying these associations.
